# Forgiveness in Romantic Relationships: The Moderating Role of Differentiation of Self in the Relationship Between Offense Severity and Post‐Offense Distress

**DOI:** 10.1111/famp.70082

**Published:** 2025-11-03

**Authors:** Agata Kasprzak, María Pilar Martínez‐Díaz, Carlos Alberto Marchena Giráldez

**Affiliations:** ^1^ Facultad de Educación y Psicología, Instituto del Perdón Universidad Francisco de Vitoria Madrid Spain; ^2^ Departamento de Psicología Universidad Pontificia Comillas Madrid Spain; ^3^ Facultad de Educación y Psicología Universidad Francisco de Vitoria Madrid Spain

**Keywords:** differentiation of self, forgiveness, moderation, offense, post‐offense distress, romantic relationship

## Abstract

This study examines the role of differentiation of self (DoS) in the forgiveness process within romantic relationships, focusing on its moderating function between perceived offense severity and post‐offense distress. The sample included 591 Spanish participants aged between 18 and 86 years (*M* = 42.66, SD = 17.06), and data were collected using validated questionnaires. Findings showed that DoS significantly moderates the relationship between offense severity and negative affect, negative cognition, and avoidance/resentment, but not with the positive dimensions of forgiveness (positive affect, benevolence, or positive behavior). Individuals with low levels of DoS experienced higher emotional distress after an offense, whereas those with high levels showed greater emotional regulation and a more realistic perception of the severity of the situation. These results highlight the importance of DoS as a regulatory resource for managing negative emotional responses but not necessarily as a promoter of prosocial attitudes or reconciliation. Results suggest that strengthening DoS in couples may help reduce the emotional burden of interpersonal transgressions. Future research could contribute longitudinal designs and dyadic analyses to capture the dynamic nature of forgiveness in romantic relationships.

## 1 Introduction

Throughout the life cycle, romantic relationships face a series of challenges and tasks that demand continuous adaptation, both individually and relationally (McGoldrick et al. 2016). In this process, individual goals and needs do not always align with those of the couple. Furthermore, due to their inherently intimate and close nature, romantic relationships often become spaces of increased vulnerability, where conflicts and associated emotional pain inevitably arise (Abreu‐Afonso et al. 2022; Metts 1994). This is especially relevant given the limited empirical research on how these processes unfold in Spanish‐speaking populations. To date, no studies have explored these forgiveness‐related processes in romantic relationships within Spanish‐speaking populations, and specifically in Spain, despite their potential clinical and relational relevance.

Conflicts or offenses within relationships may not only stem from differences in opinions or interests, but also from the breach of relational agreements or the betrayal of expectations (Fife et al. 2008; Makinen and Johnson 2006; Scuka 2015), such as infidelity, jealousy, deception, or neglect (Cameron et al. 2002; Dacka et al. 2023; Malachowski and Frisby 2015) among others. Such experiences may lead the injured party to experience a variety of emotional responses: anger or rage (Freedman and Zarifkar 2016), pain or sadness (Porter 2003), distress (Younger et al. 2004), confusion (Narváez and Díaz 2009), disappointment or a sense of betrayal (Witvliet et al. 2008). They may also elicit avoidance or distancing behaviors (Schumann and Dragotta 2021; Worthington 2002), and even desires for revenge (Jackson et al. 2019; Schumann and Dragotta 2021). When not appropriately addressed, such reactions can lead to relational deterioration and jeopardize the stability of the relationship (Fincham et al. 2006).

Conflict is not inherently harmful if managed constructively. In fact, Ogolsky et al. (2017), in their relationship maintenance model, identify forgiveness as both a threat mitigation strategy and a tool for relational enhancement. Forgiveness is a transformation process with significant potential to help individuals recover from the emotional injuries caused by painful interpersonal experiences (Enright and Fitzgibbons 2015; Fincham et al. 2007). It emerges as a key resource in overcoming offenses, closely linked to both the perceived severity of the transgression and the emotional resources available to the partners. In this regard, providing empirical evidence from the Spanish context helps expand the scope and applicability of forgiveness research beyond predominantly Anglo‐Saxon samples. To contribute to this aim, the present study examines whether differentiation of self‐moderates the relationship between perceived offense severity and both post‐offense distress and the positive dimension of forgiveness in romantic relationships, using a Spanish sample.

### 1.1 Conceptualization of Forgiveness

The process of forgiveness begins with the perception of an offense and requires that the victim recognize the experienced injustice and its intentionality (Fincham 2009). The subjective experience that follows has been described in the literature as *unforgiveness* (Worthington Jr and Wade 1999), manifested through emotional (Adams and Inesi 2016; Freedman and Zarifkar 2016; Ingersoll‐Dayton et al. 2010), cognitive (Barber et al. 2005; Nadler et al. 2008; Tripp et al. 2007; Stackhouse et al. 2018), and behavioral (Jackson et al. 2019; Lawler‐Row et al. 2008; Worthington 2002) responses following the transgression.

Many authors (Allemand et al. 2013; Fincham et al. 2004; Wade and Meyer 2009) consider forgiveness as a way to alleviate post‐offense distress, primarily by reducing negative responses such as resentment, anger, revenge, and avoidance, collectively referred to as the negative dimension of forgiveness. Furthermore, several studies (Fincham et al. 2005; Fincham and Beach 2002; Paleari et al. 2005; Wade and Worthington Jr. 2005) highlight that forgiveness can foster positive emotions, thoughts, and motivations (benevolence), comprising the positive dimension of forgiveness.

Numerous studies have demonstrated the potential benefits of forgiveness for interpersonal health (Barcaccia et al. 2018; Friedberg et al. 2009; Griffin et al. 2015; Lee and Enright 2019; Orcutt 2006), well‐being (Bono et al. 2008; Chen et al. 2019; Gismero‐González et al. 2020; Gull and Rana 2013; Toussaint and Friedman 2009), and relational quality (Gismero González et al. 2019; Wu et al. 2022). In romantic contexts specifically, forgiveness is considered a fundamental relational strategy for maintaining the relationship (Kato 2016; Kaleta and Jaśkiewicz 2024; Ogolsky et al. 2017; Waldron and Kelley 2005), as well as a mechanism to regulate emotional states and repair damage (Burnette et al. 2014), thereby contributing to relational well‐being (Fahimdanesh et al. 2020; Fincham 2000; Paleari et al. 2009). Interpersonal forgiveness in romantic relationships has been associated with indicators such as marital satisfaction, intimacy, closeness, constructive communication, and commitment (Fahimdanesh et al. 2020; Fincham and Beach 2002; Paleari et al. 2010; Wu et al. 2022).

### 1.2 Perceived Severity of the Offense in the Forgiveness Process

Within the forgiveness process, the perceived severity of the offense plays a central role. While some transgressions may be perceived as minor and are quickly resolved, others may be experienced as profound betrayals, making forgiveness significantly more difficult.

Multiple studies (Ermer et al. 2022; Fincham et al. 2005; Karremans et al. 2005; Rye and Pargament 2002) have found an inverse relationship between forgiveness and perceived offense severity. As perceived severity increases, the capacity to forgive tends to decrease. McCullough et al. (2003) suggest that in cases of highly severe transgressions, individuals may resort to avoidance or revenge as protective strategies. Other researchers point out that increased severity is associated with greater attributions of blame and responsibility, which heightens the desire for revenge (Bradfield and Aquino 1999).

In romantic relationships, serious offenses are more difficult to forgive, and perceived severity is negatively associated with forgiveness (Boon and Slusky 1997; Behrens and Kröger 2024; Morse and Metts 2011). For example, infidelity has varying impacts depending on whether it is a one‐time, without affective, or an ongoing affair with emotional attachment (Baucom et al. 2006; González Martín et al. 2011). Therefore, how a transgression is perceived influences both the emotional experience and the willingness to restore the relationship—factors that may be linked to the differentiation of self‐level of each partner.

### 1.3 Differentiation of Self

The concept of differentiation of self (DoS) refers to an individual's ability to self‐regulate emotionally. It is expressed in the degree to which a person is capable of: (a) achieving a balance between cognitive and emotional functioning, and (b) maintaining an adaptive adjustment between intimacy and autonomy in interpersonal relationships (Bowen 1978; Keller and Noone 2020; Kerr and Bowen 1988). Accordingly, it includes both an intrapsychic dimension (distinguishing emotional from cognitive processes) and an interpersonal one (preserving meaningful emotional connections with others while maintaining a clearly defined and autonomous sense of self; Bowen 1978; Keller and Noone 2020; Kerr and Bowen 1988; Skowron and Friedlander 1998).

According to Bowen (1978), the way individuals cope with stress is influenced by their level of differentiation of self, which also affects their capacity to sustain significant interpersonal relationships and manage conflict. Individuals with higher levels of DoS are more resilient to stress than those with lower levels. When faced with emotionally challenging interpersonal experiences, highly differentiated individuals are better able to regulate their emotional activation, which is associated with greater emotional maturity and interpersonal competence (Rodríguez‐González et al. 2019). As a result, these individuals function in a more autonomous and self‐directed manner, maintaining emotional balance and respecting both their own identity and that of others (Bowen 1991; Keller and Noone 2020). In contrast, individuals with lower levels of DoS tend to seek fusion, displaying a greater need for acceptance and affection from others (Kerr and Bowen 1988; Keller and Noone 2020).

### 1.4 Relationship Between Differentiation of Self and Forgiveness

DoS has been shown to facilitate individuals' capacity to sustain meaningful relationships while effectively navigating interpersonal and emotional challenges (Lampis et al. 2017). According to Baumeister et al. (1998), the emotional dimension of forgiveness is mediated by cognitive appraisal and interpretation processes. Given that DoS involves balancing emotional regulation with cognitive processes, it may facilitate individuals' capacity to recognize offenses, evaluate their impact and severity, and ultimately initiate forgiveness.

Several authors (Gordon and Baucom 1998; Thompson et al. 2005) emphasize cognitive processes like broadening perspectives, understanding the offender's viewpoint, revising beliefs, and forming realistic assumptions about oneself, others, and the relationship. These processes help the injured party gain perspective and restore safety and control. A greater ability to distinguish thoughts from feelings may support accurate offense appraisal and foster a forgiveness process that reduces post‐offense distress. Individuals with higher DoS are more likely to process offenses autonomously, assign meaning to the experience, and offer genuine forgiveness. In contrast, those with lower DoS often struggle with emotional regulation (Duch‐Ceballos et al. 2021), are more emotionally reactive (Keller and Noone 2020; Kerr and Bowen 1988), and experience offenses with greater intensity, leading to emotional fusion or cutoff, thereby prolonging the experience of post‐offense distress.

A review by Solomon et al. (2009) found that individuals with higher DoS report fewer intrusive thoughts, emotional distress, and behavioral dysfunction, as well as lower anxiety and depression. These findings align with Bowen's hypothesis of an inverse relationship between chronic anxiety and DoS: high anxiety hampers poorly differentiated individuals' coping abilities, increasing psychological and relational difficulties. Low DoS also hinders tolerance for the ambivalence inherent in relationships, which in turn may interfere with the forgiveness process. Some individuals with low DoS may avoid addressing offenses, preserving superficial harmony by minimizing the offense's significance or emotional impact, which may obstruct genuine forgiveness.

Although few studies have examined DoS and forgiveness, some report positive associations. Hill et al. (2011) highlighted how higher DoS enhances forgiveness, while lower DoS hinders it. Sandage and Jankowski (2010), in a study with 213 graduate students, found that DoS mediated the relationship between dispositional forgiveness and spiritual instability, mental health, and well‐being. Similarly, Kaleta and Mróz (2022), studying 216 university students, showed that three DoS dimensions (emotional reactivity, I‐position, and emotional cutoff) partially mediated the link between forgiveness and anxiety. Specifically, emotional reactivity and emotional cutoff mediated the link between lower levels of forgiveness and higher anxiety, while I‐position and emotional cutoff mediated the positive relationship between forgiveness and reduced anxiety. These findings suggest DoS may protect against anxiety after being hurt.

Two studies explored specific forgiveness in romantic relationships. Heintzelman et al. (2014), with 587 participants affected by infidelity, found DoS positively associated with forgiveness and moderated the relationship between trauma and forgiveness. Similarly, Dekel (2010), studying 230 women whose partners were former prisoners of war, reported that family forgiveness reduced distress among women with high levels of emotional fusion or detachment, both indicators of low DoS.

Despite the limited number of studies examining the relationship between forgiveness and DoS, existing research suggests a positive association between them in both dispositional and specific contexts. Yet the precise role of DoS remains unclear, with some studies identifying mediating effects (Kaleta and Mróz 2022; Sandage and Jankowski 2010) and other moderating effects (Heintzelman et al. 2014). Furthermore, these studies present limitations such as the reliance on university samples, a narrow focus on infidelity (Heintzelman et al. 2014), and a lack of consideration of relational factors like relationship duration and satisfaction, which constrain generalizability.

The present study aims to further knowledge in this area by analyzing the role of differentiation of self in the forgiveness process within romantic relationships in a general population sample. Specifically, it explores the potential moderating role of DoS in the relationship between perceived offense severity and post‐offense distress, as well as between perceived offense severity and the positive dimension of forgiveness. We hypothesized that differentiation of self would play a moderating role in the relationship between perceived offense severity and forgiveness (specifically in the affective, cognitive, benevolence, and resentment/avoidance dimensions, but not in the behavioral dimension). We expected this association to be stronger among individuals with low levels of differentiation of self compared to those with high levels.

## 2 Method

### 2.1 Participants

The study began with an initial sample of 906 participants. A total of 315 participants were excluded based on the following criteria: being in a romantic relationship of less than 3 months' duration; failure to complete the entire survey or all questionnaire items; and/or a score equal to or greater than 20 on the Pseudo‐Forgiveness scale of the EFI‐30. This final exclusion criterion was established following the recommendations of Enright et al. (2021), who set this score as a cutoff point to determine that individuals scoring 20 or above do not perceive a real injustice or a significant problem in the offense and, therefore, are not engaging in a true forgiveness process but rather in pseudo‐forgiveness.

A non‐probabilistic snowball sampling method was used for data collection, and the questionnaires were administered via the Qualtrics platform. The survey was initially distributed through organizations working with couples (e.g., training centers, counseling services), and additional participants were reached using the snowball sampling strategy. On the first screen of the invitation, the study requirements were presented: being in a romantic relationship, residing in Spain, and agreeing to participate voluntarily.

The final sample used for data analysis consisted of 591 participants, aged between 18 and 86 years (*M* = 42.66; SD = 17.06), including 172 men (*M* = 49.91; SD = 17.48) and 419 women (*M* = 39.57; SD = 15.92). The average duration of their romantic relationships was 18.73 years (SD = 16.30), with an average cohabitation period of 15.73 years (SD = 16.24).

Regarding parenthood, 41.6% of participants reported having no children, 7.3% reported one child, 26.9% reported two children, 13.5% reported three children, and 10.9% reported four or more.

### 2.2 Procedures

The study was approved by the Ethics Committee of Universidad Pontificia Comillas (Approval code: 2022/32). Before participation, subjects received information about the study's objectives via an informed consent form. Participation was voluntary and not financially compensated. Data were anonymized, and researchers were available throughout the process to address questions. The order of questionnaires was randomized to avoid order bias. The estimated time to complete the questionnaires was approximately 15 min. Data were collected between April 2022 and January 2023.

### 2.3 Measures

#### 2.3.1 Offense Severity and Timing

A custom‐made questionnaire was developed to assess the perceived offense severity and the time elapsed since the offense. Participants were asked to recall a real offense experienced in their current romantic relationship (the most recent, deep, and unjust one), briefly describe it (open‐ended format), rate its severity (1 = *not severe* to 6 = *very severe*), and indicate how long ago it occurred (1 = *less than a week* to 4 = *more than a year*).

#### 2.3.2 Sociodemographic Questionnaire

A custom‐made questionnaire was developed to collect demographic information (age, gender) and data related to the current romantic relationship (relationship duration, cohabitation length, number of children).

#### 2.3.3 Forgiveness

Interpersonal forgiveness was measured using the Spanish adaptation of the Enright Forgiveness Inventory (EFI‐30; Kasprzak et al. 2023). This instrument assesses specific interpersonal forgiveness by asking participants to respond in relation to a specific real‐life offense. The EFI‐30 includes 30 items across six subscales: positive affect, negative affect, positive behavior, negative behavior, positive cognition, and negative cognition. The current study used non‐inverted scores for the negative subscales (behavior, cognition, and affect), interpreting them as post‐offense distress. The Spanish version reported adequate internal consistency, with Cronbach's alpha values of 0.92 for positive affect, 0.87 for negative affect, 0.90 for positive behavior, 0.90 for negative behavior, 0.80 for positive cognition, and 0.81 for negative cognition (Kasprzak et al. 2023). In the present study, Cronbach's alphas were 0.90 for positive affect, 0.88 for negative affect, 0.84 for positive behavior, 0.87 for negative behavior, 0.74 for positive cognition, and 0.87 for negative cognition. The EFI‐30 also includes a five‐item pseudo‐forgiveness scale, which identifies incomplete or failed forgiveness processes. This scale was used as an exclusion criterion.

#### 2.3.4 Specific Offense Forgiveness

Specific interpersonal forgiveness in romantic relationships was assessed using the Spanish adaptation of the Marital Offense‐Specific Forgiveness Scale (MOFS; Kasprzak and Martínez‐Díaz 2025; Paleari et al. 2009). The scale includes 10 items measuring two dimensions: benevolence (conciliatory responses) and avoidance/resentment (vengeful and withdrawing responses). In the original study, Cronbach's alpha was 0.85 for benevolence and 0.63 for avoidance/resentment. In the present study, the alpha values were 0.63 and 0.83, respectively.

#### 2.3.5 Differentiation of Self

We used the Spanish adaptation of the Differentiation of Self Inventory–Revised (DSI‐R; Skowron and Schmitt 2003) by Rodríguez et al. (2015) to measure differentiation of self. This 26‐item instrument includes two subscales: emotional reactivity and emotional cutoff. The Spanish version showed internal consistency values of 0.85 for the total scale, 0.84 for emotional reactivity, and 0.78 for emotional cutoff. In this study, the alpha values were 0.88 (total), 0.85 (reactivity), and 0.83 (cutoff).

### 2.4 Data Analysis

Given the sample size (*N* = 591), a normal distribution of variables was assumed (Prado et al. 2015), allowing the use of parametric analyses. First, a Pearson correlation matrix was computed for the variables of interest. To assess whether differentiation of self moderated the relationship between offense severity and (a) the positive dimension of forgiveness and (b) post‐offense distress, a moderation analysis was conducted using Model 1 (simple moderation) with 10,000 bootstrapped samples and a 95% confidence interval (Hayes 2013). Based on quartiles, participants were categorized into low, medium, and high DoS. In all models, age, gender, relationship duration, cohabitation length, and number of children were included as covariates to control. SPSS version 25 and PROCESS macro v4.3 were used for all statistical analyses.

## 3 Results

Table 1 presents means, standard deviations, and score ranges for all measures used in the study.

**TABLE 1:** Descriptive analysis of instruments.

Questionnaire	Scale	*M* [range]	SD
	Time since offense	2.66 [1–4]	0.95
	Offense severity	3.74 [1–5]	1.03
EFI‐30	Positive Affect	23.88 [5–30]	5.49
	Negative Affect	11.33 [5–30]	5.39
	Positive Behavior	23.88 [5–30]	4.62
	Negative Behavior	11.74 [5–30]	5.15
	Positive Cognition	26.84 [5–30]	3.57
	Negative Cognition	7.27 [5–30]	3.86
	Pseudo‐Forgiveness	11.30 [5–30]	4.19
MOFS	Avoidance/Resentment	23.75[6–30]	5.29
	Benevolence	17.56 [4–20]	2.97
DSI‐R	Emotional Reactivity	3.29 [1–6]	0.84
	Emotional Cutoff	4.55 [1–6]	0.74
	Differentiation of Self	3.92 [1–6]	0.68

Abbreviations: DSI‐R, Differentiation of Self Inventory–Revised; EFI‐30, Enright Forgiveness Inventory; M, mean; MOFS, Marital Offense‐Specific Forgiveness Scale; SD, standard deviation.

Table 2 displays the bivariate correlations among the primary study variables. Significant negative relationships were found between offense severity and post‐offense distress variables (negative affect, negative behavior, negative cognition, and resentment), and significant positive relationships were observed between offense severity and some variables of the positive forgiveness dimension (positive behavior and benevolence). Differentiation of self showed significant correlations with all study variables.

**TABLE 2:** Correlations between forgiveness and study variables.

	1	2	3	4	5	6	7	8	9
1. Offense severity									
2. Differentiation of Self	−0.19**								
3. Positive Affect	−0.054	0.20**							
4. Negative Affect	0.21**	0.30**	−0.74**						
5. Positive Behavior	−0.13**	0.20**	0.55**	−0.50**					
6. Negative Behavior	0.16**	−0.28**	−0.53**	0.60**	−0.67**				
7. Positive Cognition	−0.05	0.22**	0.55**	−51**	0.50**	−0.43**			
8. Negative Cognition	0.14**	−0.30**	−0.49**	0.57**	−0.41**	0.50**	−0.71**		
9. Avoidance/Resentment	0.21**	0.40**	−0.36**	0.49**	−0.30**	0.43**	−0.38**	0.44**	
10. Benevolence	−0.15**	0.18**	0.34**	0.32**	0.34**	−0.28**	0.38**	−0.33**	−0.46**

** *p* < 0.01.

### 3.1 Moderating Role of Differentiation of Self

The results revealed a significant moderating effect (*p* < 0.05) of differentiation of self (DoS) in the relationship between offense severity and negative affect, *β* = −0.76; SE = 0.34; *t* = −2.22; *p* = 0.027 [1.4391–0.866], negative cognition, *β* = −0.56; SE = 0.25; *t* = −2.33; *p* = 0.020 [−1.0783 to 0.092], and avoidance/resentment, *β* = −0.81; SE = 0.33; *t* = −2.49; *p* = 0.013 [1.4501–0.1704]. DoS was not found to be a significant moderator in the relationships between offense severity and the remaining dimensions (positive affect, positive behavior, positive cognition, benevolence, and negative behavior).

**FIGURE 1:**
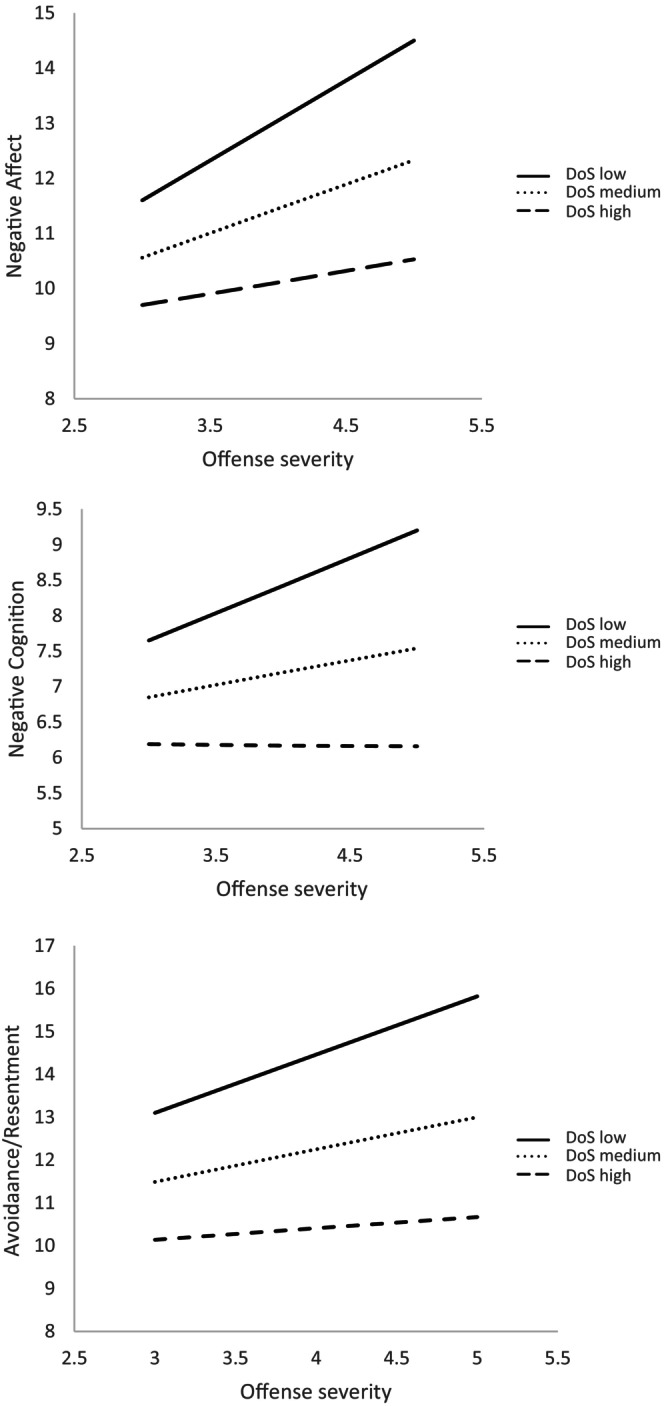
Moderating effect of differentiation of self on the relationship between offense severity and negative affect.

More specifically, as illustrated in Figure 1, the moderating effect of DoS on the relationship between offense severity and negative affect was significant among individuals with both low, *β* = 1.45; SE = 0.34; *t* = 4.25; *p* = 0.000 [0.7815–2.1250], and moderate levels of DoS, *β* = 0.89; SE = 0.23; *t* = 3.99; *p* = 0.000 [0.4511–1.3241]. However, this effect was not observed among individuals with high levels of DoS, *β* = 0.42; SE = 0.30; *t* = 1.38; *p* = 0.165 [−0.1786 to 1.0148]. More specifically, the Johnson–Neyman technique indicated that DoS ceased to moderate this relationship beyond a score of 4.42 on the DoS scale.

Regarding the relationship between offense severity and negative cognition (see Figure 1), a moderating effect of differentiation of self (DoS) was observed among individuals with low levels of DoS, *β* = 0.78; SE = 0.25; *t* = 3.12; *p* = 0.002 [0.2882–1.2671], as well as those with moderate levels, *β* = 0.34; SE = 0.16; *t* = 2.12; *p* = 0.020 [0.0253–0.6614]. No such moderating effect was found among participants with high levels of DoS, *β* = −0.02; SE = 0.22; *t* = −0.08; *p* = 0.946 [−0.4518 to 0.4178]. The Johnson–Neyman technique indicated that differentiation of self ceased to act as a moderating variable beyond a score of 4.00 on the DoS scale.

Lastly, concerning the interaction between offense severity and avoidance/resentment (see Figure 1), a moderating effect of DoS was also found among individuals with low, *β* = 1.36; SE = 0.33; *t* = 4.20; *p* = 0.000 [0.7242–1.9954], and moderate levels of DoS, *β* = 0.76; SE = 0.21; *t* = 3.61; *p* = 0.003 [0.3459–1.719], but not among those with high levels of DoS, *β* = 0.26; SE = 0.29; *t* = 0.91; *p* = 0.366 [−0.3043 to 0.8249]. According to the Johnson–Neyman analysis, the moderating role of DoS ceased to be significant beyond a score of 4.32.

To facilitate interpretation of the main effects, a baseline model without the interaction term is provided in Table S1.

## 4 Discussion

The findings of the present study offer a new perspective on the role of DoS in the forgiveness process within romantic relationships, particularly in its moderating role in the relationship between perceived offense severity and post‐offense distress. The results demonstrate that DoS significantly moderates the relationship between offense severity and negative affect, negative cognition, and avoidance/resentment among individuals with low and moderate levels of DoS. This suggests that lower levels of differentiation impair emotional and cognitive regulation in romantic contexts, making it more likely for distress to persist after an offense. In this regard, Bowen's theory describes how individuals with lower differentiation exhibit a greater need for fusion and seek acceptance and affection from others or distance and detachment (Keller and Noone 2020; Kerr and Bowen 1988).

The findings of this study suggest that individuals with lower levels of DoS tend to become so emotionally entangled in the situation that their behavior is driven by what is happening in the relationship, without being able to distinguish between emotional and intellectual processes. Previous research has shown that individuals with low DoS experience higher levels of anxiety (Xue et al. 2018) and daily stress (Murdock and Gore Jr. 2004). Consequently, when faced with an offense, these individuals are more likely to struggle with the anxiety and tension such events provoke, increasing the likelihood of experiencing unforgiveness—characterized by anger, rumination, avoidance, and resentment toward the offender. These individuals also tend to remain fixed on negative affect and cognition, which hinders their ability to process the offense and engage in a meaningful forgiveness process. Additionally, people with low DoS tend to be more emotionally reactive (Kerr and Bowen 1988), leading them to experience offenses more intensely, potentially resulting in emotional fusion or emotional cutoff, thereby prolonging post‐offense distress.

On the other hand, the results of this study suggest that higher levels of DoS enable individuals to better regulate their emotional and cognitive responses following an offense in a romantic relationship. As Lampis et al. (2017) have shown, DoS promotes the establishment of healthy relationships even in the face of adverse experiences. People with high DoS regulate their emotional arousal more effectively during interpersonal conflict (Rodríguez‐González et al. 2019), which may explain why they are better able to balance emotional and intellectual processes in response to an offense. This balance allows them to gain a realistic understanding of both the event and their internal experience, resulting in post‐offense responses that are more attuned to the actual severity of the offense. In this sense, DoS is the ability to differentiate thoughts from feelings (Bowen 1978), facilitates realistic perception and acknowledgment of the offense, thereby supporting the forgiveness process.

In romantic relationships, highly differentiated individuals also demonstrate greater autonomy from their partners, which enables them to distinguish the offense from the person who committed it. This ability allows them to experience and process post‐offense distress and to forgive their partner without fusing with them emotionally, thereby maintaining healthy boundaries even during moments of tension.

However, it is important to note that DoS was not found to moderate the relationship between offense severity and the negative forgiveness dimension of negative behavior. This result may be explained by the fact that DoS is more closely related to the ability to balance cognitive and emotional functioning (Bowen 1978; Kerr and Bowen 1988) than to the behavioral component of forgiveness.

In contrast, the present study found no moderating effect of DoS in the relationship between offense severity and the positive dimensions of forgiveness, such as positive affect, benevolence, or positive behavior. This suggests that the influence of DoS may lie more in reducing post‐offense distress than in promoting positive forgiveness responses. This difference may be explained by the fact that people with low levels of DoS tend to have greater difficulty maintaining a realistic and adjusted view of the offense and the relational situation, which is associated with a greater intensity of negative emotions and ruminative thoughts after the transgression (Kaleta and Jaśkiewicz 2024). In contrast, the more positive dimensions of forgiveness, such as benevolence, seem to be more related to relational factors such as relationship satisfaction, empathy, positive communication, and trust (Fincham et al. 2006)—aspects that were not evaluated in the present study. Moreover, the initial phases of the forgiveness process, such as the reduction of negative affect and avoidance behaviors, may be more directly influenced by individual characteristics such as DoS. However, the more advanced phases, such as approach behaviors and the generation of goodwill toward the offender, seem to depend on relational variables that go beyond the level of differentiation of self and that should also be considered in future research.

These findings broaden our understanding of the forgiveness process in romantic relationships and highlight the importance of DoS as a capacity for decreasing the emotional burden of a transgression, though not necessarily as a facilitator of reconciliation or prosocial attitudes following an offense.

In this regard, Kaleta and Mróz (2023) found that high levels of DoS contribute to reduced anxiety and negative emotions following an offense but are not directly associated with the promotion of positive forgiveness responses such as benevolence or reconciliation. Similarly, Telli and Yavuz Güler (2023) found that DoS is related to a lower tendency toward rumination and resentment but does not significantly predict the development of positive affect or relationship repair. These findings support the notion that DoS functions as a buffer against negative emotions, rather than as a promoter of prosocial attitudes after a transgression.

### 4.1 Implications

The results of this study have important implications for therapeutic intervention with couples, especially in contexts of conflict and forgiveness processes. First, therapists working with couples may focus on strengthening DoS, since higher levels of differentiation are associated with lower intensity of negative emotional responses to transgressions. In this context, emotion regulation techniques can help couples develop greater emotional autonomy, reducing negative cognitive rumination and resentment following an offense. In addition, training in emotional self‐regulation and relational autonomy may be beneficial, as VanBergen et al. (2021) found that DoS is a significant predictor of marital stability and relationship satisfaction.

However, given that the present study found no moderating effect of DoS on the positive dimensions of forgiveness, therapists may want to complement these interventions with strategies aimed at promoting empathy, affective communication, and the rebuilding of trust, thereby facilitating relationship restoration beyond the mere reduction of post‐offense distress.

### 4.2 Limitations and Future Directions

Although the findings of the present study expand our understanding of forgiveness in romantic relationships and underscore the importance of DoS as a moderating variable between offense severity and post‐offense distress, identifying it as a key resource for mitigating emotional burden, they should be interpreted in light of certain limitations.

First, the cross‐sectional nature of the study is a limitation. Future research would benefit from using longitudinal designs to examine the forgiveness process more accurately over time and to explore whether DoS changes over time and how it impacts relationship stability following a serious offense. Second, although the sample is large and includes individuals at different stages of the life cycle, it was collected through convenience sampling, which may slightly limit the generalizability of the findings. Third, the sample was composed predominantly of women, which could introduce gender bias in the results. However, previous meta‐analytic evidence indicates that gender differences in forgiveness tend to be small or non‐significant (Fehr et al. 2010). It would also be valuable to conduct future studies involving both members of the couple, allowing for dyadic analyses that include not only the victim's perspective but also the experiences of the partner who committed the offense.

Despite these limitations, this study makes an important contribution to the field of forgiveness in romantic relationships, particularly within the Spanish context, where empirical studies on this topic are scarce. Up to now, no previous research has investigated the role of differentiation of self in the forgiveness process in romantic relationships within the Spanish context.

## Conflicts of Interest

The authors declare no conflicts of interest.

## Supporting information


**Appendix S1:** famp70082‐sup‐0001‐AppendixS1.docx.

## Data Availability

The data that support the findings of this study are available on request from the corresponding author. The data are not publicly available due to privacy or ethical restrictions.
