# Supplementary material for: Forgiveness in Romantic Relationships: The Moderating Role of Differentiation of Self in the Relationship Between Offense Severity and Post‐Offense Distress
Source: Fam Process. 2025 Nov 3;64(4):e70082. doi: 10.1111/famp.70082 (PMC12582876; doi:10.1111/famp.70082)
Supplement: Supplementary file 1 — Appendix S1: famp70082‐sup‐0001‐AppendixS1.docx. [file FAMP-64-0-s001.docx]

**Supplemental Material**

**Table S1**

*Baseline Model*

| Predictive model of | Variables | *β* | t | R^2^ | F |
| --- | --- | --- | --- | --- | --- |
| Negative Affect | Differentiation of Self | -.27 | -6.67*** | .11 | 36.47*** |
|  | Offense severity | .16 | 3.94*** |  |  |
| Negative Behavior | Differentiation of Self | -.25 | -6.31*** | .09 | 28.51*** |
|  | Offense severity | .11 | 2.84* |  |  |
| Negative Cognition | Differentiation of Self | -.28 | -7.02*** | .10 | 30.74*** |
|  | Offense severity | .08 | 2.06* |  |  |
| Avoidance/Resentment | Differentiation of Self | -.37 | -9.78*** | .18 | 63.20*** |
|  | Offense severity | .14 | 3.54*** |  |  |
| Positive Affect | Differentiation of Self | .20 | 4.80*** | .04 | 12.38*** |
|  | Offense severity | -.02 | -.37 |  |  |
| Positive Behavior | Differentiation of Self | .18 | 4.37*** | .05 | 15.09*** |
|  | Offense severity | -.10 | -2.42* |  |  |
| Positive Cognition | Differentiation of Self | .22 | 5.28*** | .04 | 14.68*** |
|  | Offense severity | -.08 | -.19 |  |  |
| Benevolence | Differentiation of Self | .72 | 3.92*** | .05 | 14.23*** |
|  | Offense severity | -.36 | -2.79* |  |  |

*Note.* * *p* < .05. ** *p* < .01. *** *p* < .001.
